# Endoscopic extraction of a foreign body using a condom

**DOI:** 10.1055/a-2445-8468

**Published:** 2024-12-03

**Authors:** Cenqin Liu, Liansong Ye, Ping Yan, Xian Shi, Bing Hu, Yi Mou

**Affiliations:** 1 Department of Gastroenterology and Hepatology, West China Hospital, Sichuan University, Chengdu, China; 2 School of West China Medicine, Sichuan University, Chengdu, China

We report the case of a 16-year-old adolescent who was hospitalized due to a foreign body in the stomach, which was removed using a condom.

Upper gastrointestinal endoscopy showed a rectangle foreign body, about 2 × 9 cm in size and shaped like a lighter, in the stomach cavity ( Fig. 1 **a** ). We tried to grasp the head of the foreign body with a snare and remove it vertically ( Fig. 1 **b** ). However, despite repeated attempts, the lighter could not pass through the esophageal entrance.

**Fig. 1:**
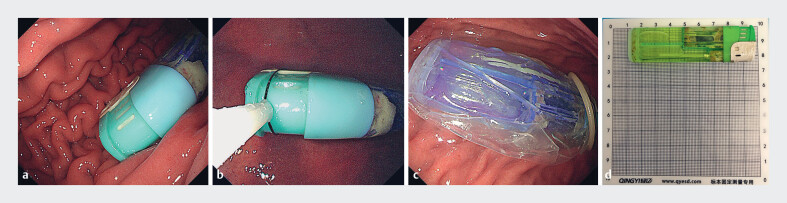
Removal of the foreign body using a condom. **a** Endoscopy showed a rectangle foreign body in the stomach cavity. **b** Attempts were made to grasp the head of the foreign body with a snare. **c** The foreign body was packed into the condom. **d** The foreign body was extracted.

Considering that a condom is a thin and flexible sheath, usually coated with water-based lubricant 1 , we decided to use this to attempt removal of the foreign body. The lighter was first placed into the mouth of the condom using the snare, and a biopsy forceps was used to clamp the opening of the condom to secure the lighter ( Fig. 1 **c** ). The lubricated condom was gently pulled through the esophageal entrance to facilitate smooth removal ( Fig. 1 **d** , Video 1 ). No complications occurred.

Endoscopic extraction of a foreign body shaped like a lighter using a condom.Video 1

The success rate of foreign body removal is affected by the anatomical spatial size of the esophageal entrance, which is also the first physiological stenosis encountered, and by compliance and coordination of the gastroscopy, all of which vary between individuals 2 . For difficult removal of foreign bodies in the upper gastrointestinal tract, the use of condoms as an additional aid seems to provide a potential and safe option for endoscopists.

Endoscopy_UCTN_Code_TTT_1AO_2AL

## Notes

Endoscopy E-Videos https://eref.thieme.de/e-videos
*E-Videos* is an open access online section of the journal *Endoscopy* , reporting on interesting cases and new techniques in gastroenterological endoscopy. All papers include a high-quality video and are published with a Creative Commons CC-BY license. Endoscopy E-Videos qualify for HINARI discounts and waivers and eligibility is automatically checked during the submission process. We grant 100% waivers to articles whose corresponding authors are based in Group A countries and 50% waivers to those who are based in Group B countries as classified by Research4Life (see: https://www.research4life.org/access/eligibility/ ). This section has its own submission website at https://mc.manuscriptcentral.com/e-videos .
